# Predictors of COVID-19 Related Health Literacy among Older People Living in Rural Areas of Indonesia*

**DOI:** 10.17533/udea.iee.v41n2e13.

**Published:** 2023-08-25

**Authors:** Fiqna Khozanatuha, Rahmi Setiyani, Lita Heni Kusumawardani

**Affiliations:** 1 RN. School of Nursing, Faculty of Health Sciences, Universitas Jenderal Soedirman, Indonesia Email: khozanatuhafiqna@gmail.com https://orcid.org/0000-0001-6232-5765 School of Nursing Faculty of Health Sciences Universitas Jenderal Soedirman Indonesia khozanatuhafiqna@gmail.com; 2 RN. MN. Assistant Professor. School of Nursing, Faculty of Health Sciences, Universitas Jenderal Soedirman, Indonesia Email: rahmi.setiyani@unsoed.ac.id. https://orcid.org/0000-0003-4107-6602 School of Nursing Faculty of Health Sciences Universitas Jenderal Soedirman Indonesia rahmi.setiyani@unsoed.ac.id; 3 RN. MN. Assistant Professor. School of Nursing, Faculty of Health Sciences, Universitas Jenderal Soedirman, Indonesia Email: litahenikusumawardani@unsoed.ac.id https://orcid.org/0000-0002-5293-2139 School of Nursing Faculty of Health Sciences Universitas Jenderal Soedirman Indonesia litahenikusumawardani@unsoed.ac.id

**Keywords:** COVID-19, aged, health literacy, rural areas, family support, Indonesia, COVID-19, anciano, alfabetización en salud, medio rural, apoyo familiar, Indonesia, COVID-19, idoso, letramento em saúde, zona rural, apoio familiar, Indonesia

## Abstract

**Objective:**

This study aims to identify predictive factors of COVID-19-related health literacy (HL) among older adults living in rural areas.

**Method:**

This study used a cross-sectional design. A total of 106 respondents participated in this study. HL was measured by using a questionnaire modified from the HLS-COVID-Q22, in addition, the scales ‘Health Information Seeking’, ‘Family’s Social Support Scale’, ‘Health Service Utilization’; and information on some socio-demographic variables was also obtained. A multiple linear regression model was used to identify the predictors of HL.

**Results:**

About two-thirds of the respondents had a moderate level of HL (63.2%). Multiple linear regression analysis showed that education level, family support, information source, and gender were significant predictors for HL (*p*<0.01).

**Conclusion:**

HL literacy was better among males, highly educated older people, media users, and those with adequate family support. This study provided insight for nurses and healthcare professionals to pay greater attention to vulnerable groups of older people (ie. female gender and those with less formal education) as well as involve family members in education or health promotion activities and use easily accessed media, such as television and radio.

## Resumen

**Objetivo:**

Este estudio tiene como objetivo identificar los factores predictivos de alfabetización en salud (AS) relacionados con el COVID-19 en los adultos mayores indonesios que viven en zonas rurales.

**Métodos:**

Este estudio utilizó un diseño de corte transversal. Un total de 106 encuestados participaron en este estudio. La AS relacionada con el COVID-19 se midió con el cuestionario HLS-COVID-Q22, además se utilizaron las escalas 'Búsqueda de información sanitaria', 'Escala de apoyo social de la familia' y 'Utilización de servicios sanitarios'; también se obtuvo información de algunas variables sociodemográficas. Se utilizó un modelo de regresión lineal múltiple para identificar los predictores de la AS.

**Resultados:**

Alrededor de dos tercios de los encuestados tenían un nivel moderado de AS (63.2%). El análisis de regresión lineal múltiple mostró que el menor nivel educativo, el apoyo familiar, la fuente de información y el sexo eran predictores significativos de la AS (*p*<0.01).

**Conclusión:**

La AS fue mejor entre los varones, las personas mayores con alto nivel educativo, los usuarios de los medios de comunicación y aquellos con un apoyo familiar adecuado. Este estudio sugirió que las enfermeras y los profesionales de la salud deben prestar más atención a los grupos vulnerables de personas mayores (especialmente las mujeres y las personas con poca educación formal). También mostró la importancia de involucrar a los familiares en las actividades de educación y promoción de la salud, y de usar medios de comunicación accesibles, como la televisión y la radio.

## Resumo

**Objetivo:**

Este estudo tem como objetivo identificar preditores de alfabetização em saúde (AS) relacionados ao COVID-19 em idosos indonésios que vivem em áreas rurais.

**Métodos:**

Este estudo utilizou um desenho transversal. Um total de 106 entrevistados participaram deste estudo. A AS relacionada à COVID-19 foi medida com o questionário HLS-COVID-Q22, além das escalas 'Pesquisa de informações em saúde', 'Escala de apoio social familiar' e 'Uso de serviços de saúde'; também foram obtidas informações sobre algumas variáveis sociodemográficas. Um modelo de regressão linear múltipla foi usado para identificar os preditores de HL.

**Resultados:**

Cerca de dois terços dos entrevistados apresentaram nível moderado de LH (63.2%). A análise de regressão linear múltipla mostrou que menor escolaridade, apoio familiar, fonte de informação e gênero foram preditores significativos de HL (p<0.01).

**Conclusão:**

A alfabetização em LH foi melhor entre os homens, idosos altamente educados, usuários de mídia e aqueles com suporte familiar adequado. Este estudo forneceu ideias para enfermeiros e profissionais de saúde prestarem mais atenção a grupos vulneráveis de idosos (ou seja, mulheres e pessoas com menos educação formal), bem como envolver os familiares em atividades de educação ou promoção de saúde e usar mídias de fácil acesso, como televisão e rádio.

## Introduction

COVID-19 pandemic has been a major global health problem worldwide. There have been about 6 million confirmed cases in Indonesia from January 2020 to May 2022.^1^ Compared to other age groups, older adults have the highest risk of hospitalization and death because of COVID-19 infection. The risks were 5 and 65 times, respectively, higher in 65 to 74-year-olds compared to 18 to 29-year-olds.^2^ In Indonesia, the COVID-19 mortality rate for older people has reached 17.68%.^3^ The increased vulnerability to coronavirus among older adults tends to be related to biological changes in the immune system that occur with aging, which are associated with age-related illnesses and susceptibility to infectious diseases.^4^ Therefore, it is very plausible to demand older people adhere to COVID-19 precautionary measures to a greater degree than younger people.^5^

Health literacy (HL) is expected to have a critical role in adopting the recommended actions. HL is defined as “the motivation, knowledge, and competence used to access, understand, appraise, as well as apply health information, and make health-related decisions”.^6^ A study suggested that HL was associated with preventive health behaviors among older people.^7^ Furthermore, it was found to be a significant determinant of preventive behaviour and awareness of the risk of COVID-19 infection.^8^ Despite its importance to individual health and well-being, HL has been underestimated during the COVID-19 pandemic.^9^ Older people are among the population groups who are most likely to experience low HL. A study demonstrated that about half of the older people had low HL.^10^ Similarly, another study identified insufficient HL in 56.1% of older people at the time of the pandemic.^11^

Few studies about HL among older people have been conducted in Indonesia. These studies, however, focused on chronic diseases context such as hypertension and diabetes mellitus.^12^^,^^13^ Health literacy of chronic diseases would be very distinct from infectious diseases like COVID-19.^14^ Further, previous studies on COVID-19 related HL worldwide mainly focused on young adult or general population^15^^-^^18^^),^. Studies specified to older adults population, however, remain limited ^11^^,^^19^. 

A slightly lower proportion of older adult in Indonesia lives in rural (48.4%) compared to urban areas (51.6%).^20^ Rural older people, however, are often considered a vulnerable and disadvantaged group since they were less likely to utilize healthcare services than their urban counterparts.^21^ Most older people in rural areas also had low (inadequate and problematic) health literacy.^22^ Even though there are few studies examined COVID-19 HL among older people, but these studies concerned those living in urban or city areas.^11^^,^^19^ To date, there is no study focused on older people population living in rural areas. 

Various models have been developed to depict factors related to HL. For example, ‘Sorensen’s Model of Health Literacy’ focuses on health literacy in non-healthcare settings.^23^ According to the model, situational factors, such as social support, family and peer influences, and media use, influence HL. Whereas the ‘RTI Health Literacy Skills Framework’ describes health literacy as “a dynamic characteristic that may be influenced by patients’ experiences from their engagement with health services”.^24^ During COVID-19 pandemic, social support and access healthcare services, however, has been significantly affected.^25^^,^^26^ Health information seeking was also significantly changed during the pandemic.^27^ Considering these major changes, therefore, it is valuable to examine how social support, access to healthcare services, and access to health information might relate to health literacy during the pandemic. 

Examining COVID-19 related HL and its predictive factors among rural older people could provide new understanding and help to fill the gap in the literature. Outcome of this study, thus can guide health workers to develop strategies to enhance older people’s compliance with preventive measures. This study aimed to determine predictors of health literacy with respect to the risk of COVID-19 infection among older adults living in rural areas.

## Methods

*Study design.* A cross-sectional survey was carried out in December 2020 among older rural residents in Ambar Ketawang Village, Sleman Regency, Yogyakarta Province of Indonesia. Yogyakarta has the highest percentage of the older people population in Indonesia.^20^

*Sample.* The sample size was determined by using a formula to estimate the population proportion with specified absolute precision.^28^ The anticipated population proportion was 50%, with a confidence level of 95%, and absolute precision of 10% resulting in 96 respondents as a minimum sample size. An additional 10% of respondents were included to prevent loss. Respondents were recruited by using the consecutive sampling method which is enrolling every single participant who meets inclusion criteria until reaching desired sample size. To be eligible, the respondents had to be aged 60-75 years, have normal cognitive function or mild impairment which demonstrated by the Short Portable Mental Status Questionnaire (SPMSQ) score of ≤ 4,^29^ be literate, and live in extended family households. Respondents were excluded if they had mental disorders, severe vision, or hearing problems. One hundred and six respondents were proportionally drawn from the six hamlets in the Ambar Ketawang village. The selection of respondents followed the recommendation of the local health authority, which provided a list of individuals who met the inclusion criteria. A total of 106 respondents were contacted and agreed to participate in this study. 

*Data collection.* Data were collected either through in-person or telephone structured interviews to standardize the data collection. Following the recommendation of the local health authority, for an in-person meeting, the duration was limited to 30 minutes for each respondent and physical distancing and protective equipment were applied during the contact. Health literacy is the dependent variable in this study. Independent variables include health information source, family support, healthcare services utilization, and sociodemographic variables. Data were collected using questionnaire comprised of five parts: health literacy, health information source, family support, healthcare service utilization, and sociodemographic. *(i) Health literacy*. The HL was measured using the modified HLS-COVID-Q22 questionnaire.^15^ The original version contains 22 items and used a 4-point rating scale on four domains: accessing, understanding, appraising, and applying health information regarding the coronavirus. In the present study, the questionnaire was translated into Bahasa Indonesia and then translated back into the original language. No significant discrepancies between the original and the translated version were found. A pilot study was conducted on 30 older people who were excluded from the main study sample to measure the reliability of the instrument. With regard to internal consistency, nearly all item-total correlations were more than 0.374, indicating good internal consistency. However, three items were removed due to their low total correlation. The Cronbach's alpha was 0.819. The final adapted version consisted of 19 items with a score ranging from 19-76. A higher score means a higher HL. Category: Inadequate ≤50% (≤47), moderate >50%-<66% (48-56), sufficient ≥66% (≥57);^22^ *(ii) Health information source*. It consisted of a single item adapted from the ‘Health Information Seeking’ measures.^(.)^ The respondents were asked where they mostly looked for information about COVID-19 during the pandemic by selecting one of the following categories: 1) family, friends, and neighbors; 2) healthcare professionals; 3) traditional media, such as television, radio, newspapers, magazines; and 4) digital media, such as the internet and social media. This variable was then further categorized into either personal (e.g., family, friends, neighbors, and healthcare professionals) or media (traditional media and the internet); *(iii) Family support*. The family support questionnaire items were derived from the ‘Family’s Social Support Scale’ which was adapted for the circumstances of this study.^31^ The original instrument was developed based upon four social support dimensions: emotional, instrumental, appraisal, and informational support, and comprised 32 question items using a 5-point Likert scale. The adapted questionnaire consisted of 17 items and used the 4-point Likert scale instead of 5 to prevent respondents from selecting a “neutral” response. A pilot test of 30 respondents showed the reliability of the questionnaire was acceptable with Cronbach's alpha coefficient of 0.874. The item-total correlations were nearly all positive and more than 0.374. The internal consistency of the questionnaire was improved by the deletion of three items. The final adapted questionnaire comprised 14 items. The total score ranges from 14-56, where a higher score would indicate higher support; *(iv) Healthcare services utilization*. The questions were derived from the ‘Health Service Utilization’ questionnaire which was adapted for the circumstances of this study.^32^ The original scale consisted of 8 items rated on a Guttman scale. A positive response would be counted as one point and a negative response as zero. After a pilot testing, 4 items were deleted due to their low total correlation (less than 0.374). The Cronbach’s alpha value was 0.734, demonstrating good internal consistency. The final questionnaire consisted of four items. The score ranges from 0-4, with a higher score meaning higher utilization; and, *(v) Sociodemographic*. The sociodemographic variables of the study’s population consisted of four items with open-ended questions and multiple-choice responses, including age, gender, education level, and income.

*Data analysis.* Data processing was performed by using the IBM SPSS version 25.0 software. Data were analyzed using descriptive statistical methods and multiple linear regression. Descriptive statistics were employed to characterize the respondents’ demographic profiles and the study’s variables. To determine the factors that influence the respondents’ HL, multiple linear regression was used. 

*Ethical considerations.* This study was approved by the Research Ethics Committee of the Faculty of Health Sciences, Jenderal Soedirman University, Indonesia, on 4 December 2020 with registration number 233/EC/KEPK/XII/2020. Respondents were treated in accordance with the tenets of the Declaration of Helsinki. Prior to participation, respondents were explained the aim and nature of this study, then signed an informed consent form if they agreed to participate. Ethical principles including autonomy, privacy, dignity, confidentiality, and anonymity were ensured throughout the study. 

## Results

A total of 106 older people participated (Table 1). The mean age of our respondents was 67.1 years (±4.91). About two-thirds of them were female (65.1%), had low education levels (junior secondary education or lower) (60.4%), and had low income (65.1%).

**Table 1:** Demographic characteristics of respondents (*n*=106)

Variables	Value
Age (years); mean (SD)	67.1 (4.91)
Gender; *n* (%)	
Male	37 (34.9)
Female	69 (65.1)
Education level; *n* (%)	
Low (junior secondary school or lower)	64 (60.4)
High (senior secondary school or college)	42 (39.6)
Income; *n* (%)	
Low (below 129 USD per month)	69 (65.1)
Standard (≥ 129 USD per month)	37 (34.9)

The result showed that almost two-thirds of the respondents had a moderate level of HL (63.2%), with a mean score of 51.36 (±5.970). The family support score was 38.80±5.36, which was considered moderate relative to its possible score range (14-56). A different result was shown by the healthcare service utilization variable with an average score of only 1.75±1.53, which is at a slightly lower end of its possible score range (0-4). Regarding information sources, most respondents accessed COVID-19-related information from the media, primarily the traditional ones, such as TV, radio, newspapers, and magazines (53.8%) (Table 2).

**Table 2:** HL, information access, family support, and healthcare services utilization (*n*=106)

Variables	Value
HL scores); mean (SD)	51.36 (5.97)
HL levels; *n* (%)	
Inadequate	21 (19.8)
Moderate	67 (63.2)
Sufficient	18 (17.0)
Information source; *n* (%)	
Family, friends, neighbors	28 (26.4)
Healthcare professionals	2 (1.9)
Traditional media (TV, radio, newspapers, magazines)	57 (53.8)
Digital media (the internet, social media)	19 (17.9)
Family support; mean (SD)	38.80 (5.36)
Healthcare services utilization; mean (SD)	1.75 (1.53)

*Range of scores for HL 19-76; family support 14-56; healthcare service utilization 0-4

Pearson correlation coefficient and independent t-test were used to determine the relationships among studied variables with the mean of HL. The result showed that family support and healthcare service utilization were significantly and positively correlated with HL (*p*<0.001 and *p*<0.01 respectively). Older people who accessed health information from media also had a better HL than those who received it from family, friends, neighbors, and healthcare personnel (*p*<0.01). All the demographic variables, except for age, showed a significant relationship with HL (Table 3).

**Table 3:** The association between HL scores and information access, family support, healthcare service utilization, and demographic characteristics (*n*=106)

Variable	Health Literacy Scores
	mean (SD) or r
Information source	
Personal (family, friend, neighbors, & healthcare professionals)	48.53 (5.58)^b,^**
Media (traditional media & digital media)	52.47 (5.77)
Family support	0.467^a,^***
Healthcare services utilization	0.257^a,^**
Age (years)	-0.051^a^
Gender	
Male	53.08 (4.83)^b,^*
Female	50.43 (6.33)
Education level	
Low (junior secondary school or lower)	48.67 (5.63)^b,^***
High (senior secondary school or college)	55.45 (3.74)
Income^b^	
Low (below 129 USD per month)	49.48 (5.84)^b,^***
Standard (≥ 129 USD per month)	54.86 (4.473)

^*a*^ *Analyzed using Pearson correlation,* ^*b*^ *analyzed using independent t-test*, *p<0.05, **p<0.01, ***p<0.001

The predictors of HL are presented in Table 4. Out of the four models, all significant predictors remained in model 4. In this model, accessing health information from media, higher family support, male gender, and higher education were significant predictors of better HL. The adjusted R^2^ of the 4^th^ model was 0.458, indicating that 45.8% of the variance observed in HL can be explained by independent variables including information source, family support, gender, and education.

**Table 4:** Multiple linear regression analysis of the predictors of HL

Variable	Model 1	Model 2	Model 3	Model 4
Information source (ref category: media)	-0.255**	-0.255**	-0.246**	-0.242**
Family support	0.299***	0.300***	0.313***	0.330***
Healthcare services utilization	0.071	0.071	-	-
Age	-0.006	-	-	-
Gender (ref category: male)	-0.215**	-0.214**	-0.218**	-0.228**
Education (ref category: high education)	-0.285**	-0.284**	-0.302**	-0.364***
Income (ref category: standard income)	-0.126	-0.127	-0.126	-
*R* ^*2*^	0.494	0.494	0.489	0.478
*Adjusted R* ^*2*^	0.458	0.463	0.464	0.458

**p<0.01; ***p<0.001

## Discussion

The present study aims to identify the factors that influence HL regarding COVID-19 among older people living in rural areas. The findings showed that accessing information from media, having better family support, being male, and attaining a higher level of education significantly influenced HL in preventing COVID-19. Health information source refers to the primary source of information accessed by older people to obtain information regarding COVID-19. In this study, those who primarily accessed the media either traditional such as television, radio, newspapers, magazines, or digital for example the internet to obtain information regarding COVID-19, were more likely to have better HL than those who received information from personal resources, such as family, friends, neighbors, and healthcare professionals. Similarly, a study by Hoa *et al*.^33^ suggested that listening to TV and radio, reading the paper, and using the internet were positively associated with HL in older people. The lower HL found among those who rely on personal resources might be because older people were suggested to stay at home and restrict their mobility as part of COVID-19 preventive measures. This situation is likely to cause them to have limited access to information. A previous study also demonstrated that older adults who have poor access to health information materials tended to show inadequate HL.^11^

Among media users, many of them accessed traditional media compared to digital media. In Indonesia, access to digital technology was lower among older age groups, as shown by lower levels of mobile phone and smartphone ownership, and usage levels of digital communications such as social media.^34^ Similarly, a study in Thailand demonstrated that senior citizens in the country had an intermediate level of internet literacy.^35^ Older people and those with low internet skills tend to find health information from conventional media instead of the internet.^30^ Older people could benefit from using the internet for various purposes, including accessing health information.^33^ It should, however, be done with caution because during the outbreak the internet and social media have exploded with inaccurate information and conflicting messages about COVID‐19.^36^

Family support was also found to be a significant predictor of older people’s HL. Older people that had higher family support were likely to demonstrate higher HL. This finding is consistent with a previous study, which indicated that older people that receive high-level social support from family members were more likely to have proficient HL.^11^ Generally, social support can come from different sources. In Indonesia, family members, particularly adult children are the main source of social support for older people.^37^

The critical role of the family in an individual’s HL has also been suggested in a study by Edwards *et al.*^38^ The study proposed the concept of ‘distributed health literacy’ which believes that HL is distributed through social networks, including families. The family acts as HL mediators as they pass their HL skills and provide support to the individual to become more health literate.^38^ This role has grown in importance in this digital age. Many older people experience difficulties in accessing information due to their limited digital skills or ability to evaluate the accuracy of the information obtained. Thus, the family may bridge the “digital divide” by facilitating access and usage of health information technology, teaching skills, and acting as online delegates.^39^

The interesting finding in this study was that even though family support was positively correlated with health literacy, older people who depend on personal resources, including family members, had a lower health literacy compared to those who used media to get information. The possible explanation is that family members can be a good source of information, however, their understanding of Covid-19 might be quite limited. Family support might be not expressed by giving older people information, but instead, giving access to the media.

Gender and education level were two demographic characteristics that significantly influence older people’s HL. In this study, those with higher education levels tended to have better HL than their counterparts. A similar finding has been indicated in different studies.^10^^,^^40^^,^^41^ Pechrapa *et al.*,^11^ however, reported no differences in HL between individuals who have attended primary school and those with better levels of education. Nevertheless, better-educated people tended to have more access to health information, better communication with healthcare providers and better skills to find out health information.^10^

The findings of this study also demonstrated that the male respondents had better HL than their female counterparts. However, there is conflicting evidence on whether gender relates to HL and reported that male older people had better HL.^41^ Still, a study by Ansari *et al.* found that female older people had better HL.^40^ Meanwhile, Pechrapa *et al.* ^11^ and Nezafati *et al*. ^10^ both found that gender was not associated with HL. In Indonesia, male older people tended to have a better education level than female older people.^21^ This fact might help to explain why they have better HL. Due to the inconsistent findings in prior studies, the role of gender and education in older people’s HL warrants further investigation.

### Limitations

One of this study’s limitations is that the number of respondents was relatively small for a survey-based study due to the implementation of community activity restrictions in some areas during the pandemic. Secondly, this study only investigated a few factors and other unmentioned factors that might contribute to COVID-19-related HL in rural older people. Thirdly, the present study was conducted only in a single region in Indonesia, and thus generalizations should be done carefully due to possible sampling bias. Lastly, the test-retest reliability of the questionnaire was also not conducted in the present study. The local health authority did not allow the investigators to re-visit the respondents to minimize the COVID-19 spread. Despite its limitations, our study is still beneficial as it is one of the very few studies to have investigated COVID-19-related HL of older people in Indonesia context.

## Conclusion

Older adults who accessed health information from media, had higher family support, are male in gender, and attained higher educational levels had better HL related to COVID-19 prevention. This study provides insight for nurses and healthcare professionals to pay greater attention to vulnerable groups of older people (female gender and those with fewer years of formal education), to the involvement of family members in education or health promotion activities that target older people, and to provide information with media that are easily accessed by older people, such as television and radio.
